# On the Nature, Cause, and Treatment of Cholera

**Published:** 1854-11

**Authors:** David Lewis

**Affiliations:** Lieentiate of the Royal College of Physicians, London; Physician to the Royal General Dispensary, Aldersgate-street


From the Lancet.

*On the Nature, Cause, and Treatment of Cholera.* By David Lewis, M. D., Licentiate of the Royal College of Physicians, London; Physician to the Royal General Dispensary, Aldersgate-street.

In the midst of so many specifics for the cure of cholera, and and such numerous theories to explain its cause, I venture to submit the following observations to notice with considerable diffidence. But I am emboldened to speak plainly. because to the best of my belief, I speak the truth—a belief founded upon impartial observation, and confirmed by a lengthened experience. I have treated many hundreds of cases of diarrhœa according to the methods generally reported to succeed, and the result of my experience is, that two only of the various plans avail to cure—viz.: the employment of alkalies, or the stronger acids. But these plans appear to fail so seldom, that I now invariably prescribe them with perfect confidence. But how can remedies, so opposed in all their characters, produce, when administered, similar effects? What is their action?

I believe the almost universal cause of diarrhœa to be an excess of acidity in the stomach and intestines, and I believe that alkalies act by neutralizing, and the mineral acids by destroying, this morbid condition. That diarrhœa is so generally caused by the presence of of a morbid material, of an acid character, I think is proved by the following facts:—A gentleman of high standing in society partook heartily of some cherry-pie for supper; he was attacked with diarrhœa in the night, and died of cholera next day. Another gentleman being in delicate health, drank two glasses of champagne at dinner; he was attacked with diarrhœa in the night, and died of cholera in nine hours. Another man drank a quart of sour beer, was soon attacked with diarrhœa, and died of cholera in twelve hours.

Now I extend this theory to the cause of cholera, and employ either of the above means for its cure, and the uniform success of my treatment strengthens my confidence in the strength of my theory. I believe, then, the cause of cholera to be some material of an acid nature, or possessing acid properties, and that by virtue of this character, it acts so as to produce cholera, and destroy life. This acid can be neutralized by akalies, or destroyed by stronger acids, even as uric acid can be neutralized by alkalies, or broken up by nitric acid. This theory I believe to be true, the facts upon which it is founded I know to be incontrovertible, and with regard to both, I would say, in the words of Horace, to those who hesitate to believe,

"Si quid novisti rectius istis,
Candidus imperti; si non, his utere mecum."

The following list includes the principal modes of treatment hitherto recommended:

Calomel, in large doses, and in smaller and frequent doses; calomel with opium; opium with large doses; alcoholic liquids; camphor and musk; acetate of lead; sulphate of copper; vegetable astringents; quinine; arsenic; iron; the saline plan, as recommended by Dr. Stevens; the tartarized-anatomy plan, as recommended by Dr. Billing; emetics; the free administration of cold water and ice; the cold bath; the application of the wet sheet; injunction of saline or other fluids into the veins; bleeding; electricity.

Calomel, accroding to my experience, both in large and small doses, has been totally worthless. Before the collapsed state ether remedies are more efficient in arresting the progress of the disease, and in the collapsed state, when the process of absorption has ceased, calomel may be given in spoonfuls without any effect. I look upon the treatment of cholera in the early stages by calomel as only tampering with human life, whilst yet the disease can be arrested to a certainty by other means. Practitioners examine the alvine evacuations, and finding deficiency of bile, immediately throw in calomel, until the secretion of the liver is established, forgetting that the biliary secretion is dependendent on the general state of health, and that the absence of bile is the consequence, and not the cause, of the disease. Calomel at the onset acts as a purgative, and relieves the stomach and bowels from any irritating matter which might tend to aggravate the diarrhœa; beyond that point it has no effect whatever on the disease. In London, calomel, given as a specific in all cases of diarrhœa, to the ill-fed and badly clothed, and those residing in pestiferous localities, be a poison as destructive as the cholera itself. In the country its effects are not so deleterious because the people are stronger and better fed. A medical practitioner at Shoreditch told me that he had given calomel "until he was sick of it," without any perceptible effect. Children and young people are not so easily killed by calomel, as the more advanced in age; nevertheless there is no reason to persevere in such a mode of treatment when there is no necessity for it.

Calomel and opium may be applicable in some cases in the early stages, but when the disease is bordering on the eclipsed state, the remedy is useless.

Opium will allay the irritation of the bowels after they have been cleared by an antacid aperient draught.

Stimulants are serviceable according to circumstances; but there is no dependence to be placed on them as specifics in the disease, with the exception of ammonia.

The acetate of lead, the sulphate of copper, &c., are poisons, and totally useless in the cure of cholera. The same remark is applicable to quinine, iron, and arsenic.

The saline plan, as recommended by Dr. Stevens, is nothing more than an indirect application of a principle to destroy the acid products of the stomach, which can only be done by either alkaline or acid remedies.

I have seen the saline treatment tried in a favorable case without any effect. The young lady died in twelve hours.

Emetics act on the principle of clearing the *primæ viæ*; there is nothing in the plan beyond relieving the contents of the stomach, which might as well be done by an aperient draught.

The free administration of cold water and cold baths do more harm than good in nine cases out of ten. In the state of collapse, when the thirst is great, cold water may be given, but I have rarely seen any permanent good effects arise from it.

The injection of saline and other fluids into the veins only answers the purhose of those who aim at notoriety at any price. Bleeding is worse than useless.

Electricity has no influence over the disease. The use that was trumpeted in all the newspapers as having occurred at the Free Hospital I happened to witness. There was no collapse,, nor any urgent symptom requiring a peculiar mode of treatment.

In September, 1849, the Cholera Committee of the Royal College of Physicians sent a list of questions to the members. One of them was the following: "Does it accord with your experience that cholera, in the stage of "serous" or watery diarrhœa, can with facility be checked? What means have you found most effectual in attaining this end?"

I have found no difficulty in arresting the serous or watery diarrhœa by either of the following plans of treatment. In the first place I assume that the disease is dependent on acidities in the stomach, and treat it accordingly. According to the first method, a powder, containing a scruple of rhubarb, and the same quantity of the carbonate of magnesia, is invariably given by me before an attempt is made to arrest the diarrhœa. Without the above purgative I found I could not depend on the astringent mixture. I have been obliged to repeat the rhubarb and magnesia more than once in excessive purging before the diarrhœa could be arrested. Instead of hurrying the diarrhœa into cholera, as has been imagined it is the only certain method of arresting the disease, since it clears the stomach and bowels from the morbific matter. After the powder has passed through the bowels I give the following mixture:—Powdered (prepared) chalk, two drachms and a half; sesquicarbonate of ammonia, sixteen grains; tincture of opium, half a drachm; compound tincture cardamons, three drachms; cinnaamon water, an ounce and a half; water, four ounces. Mix. Two tablespoonsfuls after every motion.

It is important to give a dose of the mixture after every motion, otherwise the objects of arresting the diarrhœa by neutralizing acidities in the *primæ viæ*. and quieting the bowels, will not succeed in equal ratio by giving the mixture every two or three hours. At the Islington Dispensary there were in 1849 *nine hundred registered cases of diarrhœa and incipient cholera*, which were all treated on the above plan, without a *single death*. Not a grain of calomel was given. Within the last three months three thousand cases have been treated on the same principle at the Royal General Dispensary, without *one* failure. The success of this plan has been such that I am perfectly satisfied with it; and I have no hesitation in saying, that if the diarrhœa be treated as above, by antacids and astringents, or by mineral acids, there would be but few cases of death from cholera.

The second method of treatment is by the employment of the stronger acids in moderate doses. These, I believe, act by at once destroying the morbid material, and thus produce the same results as alkalies, which neutralize it; for this purpose two drachms of diluted sulphuric acid to six ounces of water, for a mixture, of which two teaspoonfuls to be given to children after every motion, or two teaspoonfuls in the same manner to adults, seldom fail of success.

Finally, I look upon the case of cholera as a poison of an acid character, or capable of generating acids in the stomach, acting solely on the mucous membrane of the stomach and bowels, and if neutralized by carbon and carbonates, or destroyed by mineral acids, it will be rendered inert, just the same as any other poison by its antidote.

Keeping this theory in view, no one need be at a loss to treat cholera on a specific principle.

